# Giant dorsal lipofibromatosis in an infant: a case report

**DOI:** 10.1186/s12887-022-03130-7

**Published:** 2022-01-22

**Authors:** Zhiyu Li, Yuqing Zou, Guangqi Xu, Jianhai Bi, Ran Huo

**Affiliations:** 1 Department of Plastic and Aesthetic Surgery, Shandong Provincial Hospital, Cheeloo College of Medicine, Shandong University, Jinan, Shandong 250021 China; 2 Department of Plastic and Aesthetic Surgery, Shandong Provincial Hospital Affiliated to Shandong First Medical University, 250021 Jinan, Shandong China; 3 Medical Science and Technology Innovation Center, Shandong First Medical University & Shandong Academy of Medical Sciences, No. 324, Jingwu Road, Huaiyin District, 250021 Jinan, Shandong Province China

## Abstract

**Background:**

Lipofibromatosis is a rare, benign, soft tissue tumor that usually presents in children. Low incidence and lack of specificity in clinical presentation make its diagnosis difficult.

**Case presentation:**

This is a case report of a patient with a giant lipofibromatosis on the back that resembles an infantile hemangioma, which posed great difficulty in diagnosis due to atypical clinical manifestations. After the postoperative pathological and immunohistochemical examination and fluorescence in situ hybridization, the patient was finally diagnosed with lipofibromatosis.

**Conclusions:**

The incidence of fibromatosis was low. This case presents an atypical clinical manifestation since the tumor growth was on the back, and this can easily cause misdiagnosis. This case suggests that the diagnosis of lipofibromatosis depends on the pathology and fluorescence in situ hybridization.

## Background

Lipofibromatosis is a rare, benign, soft tissue tumor that develops mainly in children and is congenital in approximately one-fifth of cases [1]. It was first proposed by Fetsch in 2000 [2]. The lesions usually develop under the skin or in the soft tissues of the extremities, typically in the hands or feet, and the most common presentation is a slowly growing, mildly painful mass. Complete surgical resection, the preferred treatment option, may result in local recurrence, and no distant metastases have been reported [1]. As the disease is relatively rare, it is prone to misdiagnosis. This is a report of an atypical case of lipofibromatosis in which the diagnosis was confirmed only after detection by various modalities such as pathology, immunohistochemistry, and fluorescence in situ hybridization (FISH).

## Case presentation

**Fig. 1:**
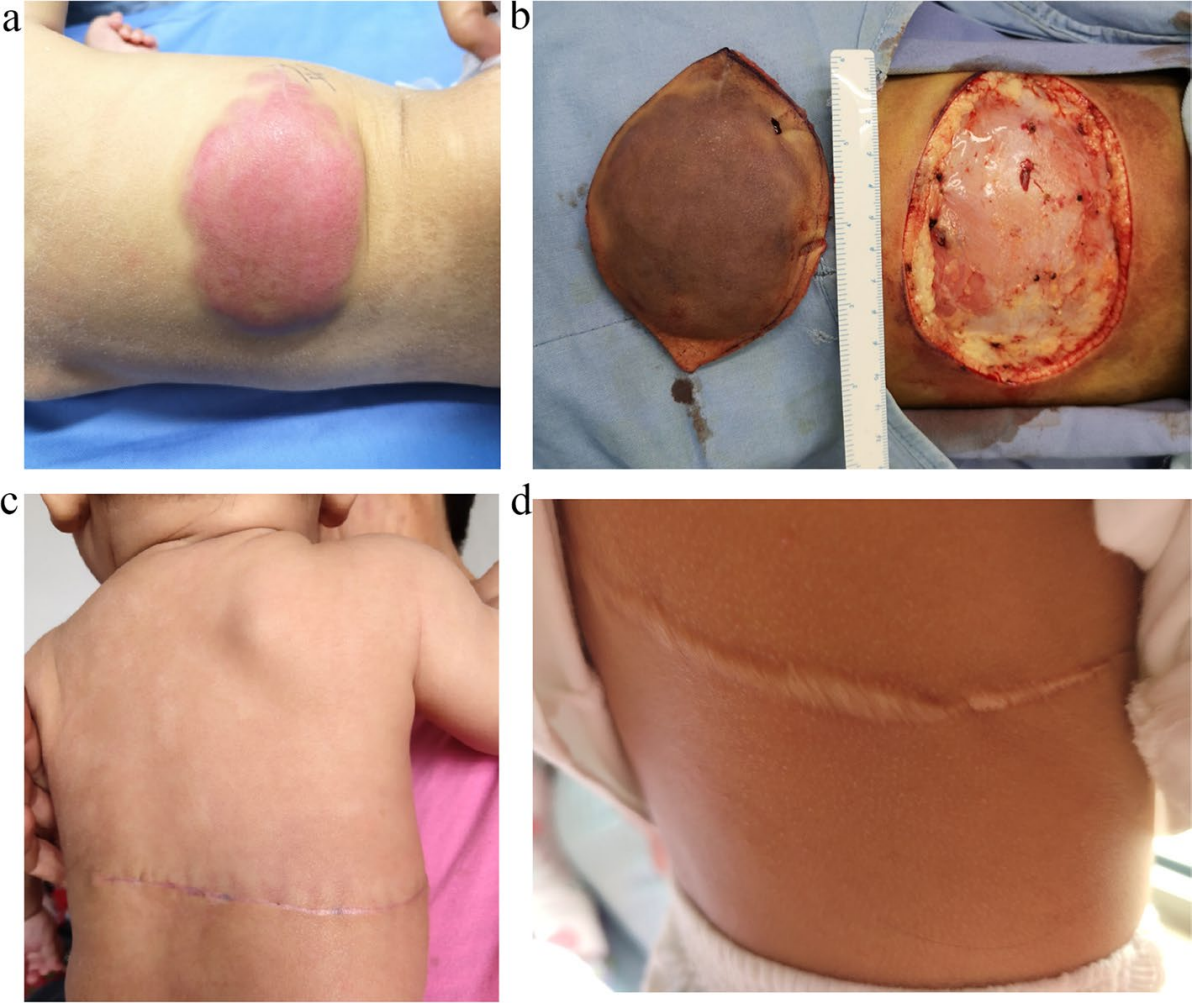
Photographs of the whole treatment process of the child. **a** The lesion at presentation of the child; **b** Intraoperative photograph of a wound up to 11 cm long after removal of the lesion; **c** Photographs of the child at the one-month follow-up after the operation; **d** Photographs taken at the child’s home one year after the operation

**Fig. 2:**
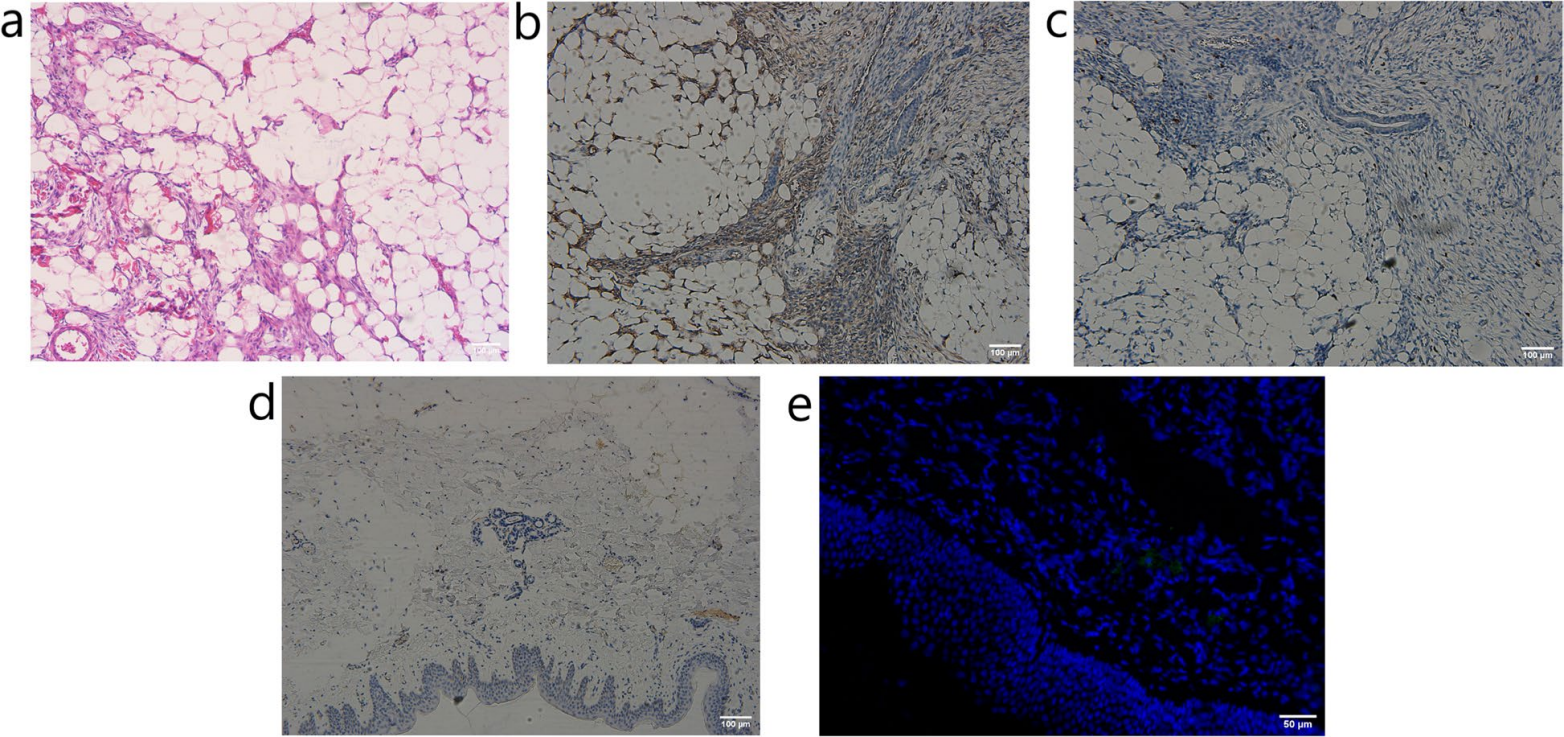
Relevant examination results. **a** Hematoxylin and eosin (HE) staining, which shows that the tumor consists of mature adipose tissue and fusiform fibroblasts; **b** Immunohistochemical staining, CD34(+); **c** Immunohistochemical staining, S-100(-); **d** Immunohistochemical staining, pan-TRK(-); **e** Fluorescence in situ hybridization (FISH) assay revealed no fusion or rearrangement of NTRK1 gene

The patient was a six-month-old female with a red patch on her back found more than 20 days after birth. Later, a gradually enlarged mass was found over the surrounding skin, which slowly increased in size as the child grew, measuring approximately 11 cm × 6 cm (Fig. 1 A). No urinary dribbling, lower extremity deformity or scoliosis, anorectal malformation or sphincter dysfunction was observed. The infant was uncooperative for neurological examination and no signs of lower limb paresis or paralysis were found. Ultrasonography revealed an infantile hemangioma. Complete blood count showed white blood cell, red blood cell, hemoglobin, and platelet levels of 7.62 × 10^9^/L, 3.98 × 10^12^/L, 112 g/L, and 436 × 10^9^/L, respectively. On laboratory tests, no significant abnormality was found. A small portion of the tumor was taken for histopathological examination, and lipofibromatosis was suspected. Upon consulting at the pathology department of another hospital, fibrous hamartoma of infancy was suspected. The mass was subsequently excised completely under general anesthesia with preoperative blood preparation (Fig. 1B, C). A fusiform incision was made 0.5 cm along the outer edge of the tumor, after which, both sides of the incision were widely free to the outer edge and sutured layer by layer. On pathological examination, spindle cell tumor was confirmed. The tumor consisted of adipose tissue and fusiform fibroblasts, which tended to be lipofibromatosis (Fig. 2 A)(Observed under the microscope OLYMPUS CX31, Olympus Corporation, Japan; photographed by the software Oplenic Pro, Hangzhou Chroma Optronics CO., LTD, China). Immunohistochemistry results included the following: CD99 (focal +), smooth muscle actin (SMA) (-), ki67 (+5-15%), CD34 (++++) (Fig. 2B), CD31 (-), ERG (-), SOX10(-), S-100 (-) (Fig. 2 C), BCL2 (-), P1H3 (+8%), P53 (-), Desmin (-) Myogenin (-), MyoD1 (-), and pan-TRK (-) (Fig. 2D)(Observed under the microscope Pannoramic MIDI, 3DHISTECH Ltd, Hungary; photographed by the software Pannoramic Scanner, 3DHISTECH Ltd, Hungary). Genetic testing showed no positive mutations in exons 18, 19, 20, and 21 of the EGFR gene. The author detected the NTRK1 gene by FISH and found no fusion or rearrangement of the NTRK1 gene (Fig. 2E). (Observed under the microscope Pannoramic MIDI, 3DHISTECH Ltd, Hungary; photographed by the software Pannoramic Scanner, 3DHISTECH Ltd, Hungary).The patient was eventually diagnosed with lipofibromatosis. One year later, the patient was reassessed, and no signs of recurrence were found (Fig. 1D), with scarring of the incision remnants. The scar will be repaired after the child grows up, and follow-up is continuing.

## Discussion and Conclusion

Lipofibromatosis is clinically characterized by painless masses with indistinct borders. It is mostly found in the extremities, especially the hands and feet, with other reports seen in the neck, orbit, back, chest, abdominal wall, and chin [3]. The age of onset ranges from 1 to 14 years [4]. The Fetsch study showed a male-to-female incidence ratio of 2.7:1 [2], and the Al-Ibraheemi statistic male-to-female incidence ratio was 2:1 [4]. Nevertheless, Boos et al. concluded that the incidence in men and women was approximately equal [3]. Most tumors are approximately 1–7 cm in diameter, with a mean diameter of approximately 2 cm [2]. Tumors exceeding 5 cm in diameter are rare [5]. The present case occurred in the back and had an appearance similar to that of an infantile hemangioma. These characteristics, along with the rapid growth of the lesion, with a length of up to 11 cm, are rare and make diagnosis difficult.

The diagnosis of lipofibromatosis relies heavily on pathology. Macroscopic findings of excised tumors are generally described as yellow, brown, or white with a firm, rubbery, or gritty texture [1]. Microscopically, tumors consist of mature adipose tissue and fusiform fibroblasts. The tumor can consist more than 50% of the adipose tissue, with adipocytes varying in size and sometimes being disorganized. Fibrous tissue element traverses the adipose tissue in the form of septa. Moreover, fibroblasts have reduced mitotic activity (0 to 3 mitotic figures per 10 high-power fields) and lack nuclear heteromorphism [1, 2, 4, 6]. Immunohistochemistry is non-specific and usually cannot be used to diagnose lipofibromatosis [1, 2]. However, immunohistochemistry can be used for differential diagnosis and to differentiate between diseases such as stromal tumors [7] and lipofibromatosis-like neural tumors (LPF-NT) [8]. Only a few studies have proposed the use of MRI for the diagnosis of lipofibromatosis [1, 3], but it does not seem widely used and cannot confirm the presence of the disease [9]. Thus, in this case, no further imaging was performed after the boundary and depth of the lesion were confirmed by ultrasound, and a direct biopsy was chosen. However, it is undeniable that MRI still has an important role in the differential diagnosis of related diseases. MRI can be performed when a patient’s condition is suitable.

The differential diagnosis of lipofibromatosis is complex and often requires differentiation from a variety of diseases, including juvenile (including congenital and infantile) fibromatosis, fibrous hamartoma of infancy (FHI), calcifying aponeurotic fibroma, lipoblastoma, lipoma, neurofibroma, and lymphatic malformation [10, 11]. Here, a few diseases more difficult to identify are discussed, namely infantile hemangioma (IH), tufted angioma (TA) or Kaposiform hemangioendothelioma (KHE), FHI, and LPF-NT.

IHs are the most common benign tumors in infancy and childhood. They may appear as erythema shortly after birth and gradually increase in size. They can grow up to nine months of age and then gradually fade away [12]. According to the parents of the child, the early presentation of the lesion was similar to that of IH and was treated with propranolol at a local hospital. However, no improvement was observed, and the tumor continued to increase in size until six months. The distinction is not accurate from medical history, clinical manifestations, and imaging, but there is a major difference in pathological manifestations. Proliferative IH is seen as large clusters of endothelial cells, irregular in shape, well-defined, swollen outward growth, and less spaced tissue [13]. Thus, IH was ruled out after biopsies were taken.

KHE on the body surface usually presents as a tough skin or subcutaneous mass with a purplish-red, nodular or plaque appearance, and poorly defined margins due to petechiae or dilated capillaries. KHE or TA can cause the Kasabach-Merritt phenomenon, a class of clinical manifestations associated with thrombocytopenia, microvascular hemolytic anemia, and consumptive coagulation dysfunction, in addition to vascular malformation. Confirmation of KHE diagnosis requires pathological evidence. KHE lesions are characterized by a large number of nodules of varying shapes and fuzzy borders infiltrating the surrounding area. The internal morphology of the nodules combines the features of both IH and Kaposi’s sarcoma. There were clusters of fusiform endothelial cells and highly tortuous, tangled microvessels, as well as a large number of poorly developed slit-like lumina filled with red blood cells. The thin-walled crescent-shaped vascular cavities and enlarged lymphatic cavities surrounding the tightly compressed lobules of the microvessels in TA lesions are also characteristic features of TA [14].

FHI usually occurs in children under two years of age, and approximately 20% of cases present as congenital lesions. A painless subcutaneous mass involving the axillae, trunk, upper arms, and external genitalia usually develops [15]. The characteristic microscopic appearance has three components, including well-defined bundles of fibrous tissue, primitive mesenchyme arranged in nests, and mature adipose tissue intimately mixed with the other components [16]. Although lipofibromatosis has similar bundles of fibroblasts and mature adipose tissue, it lacks immature mesenchymal cells. Thus, pathological examination remains the primary method of differentiation. Immunohistochemical examination may be positive for SMA. In addition, Jason et al. found EGFR exon 20 deletion or duplication mutations as a characteristic feature of FHI [17]. The present case was also tested, and no positive mutations in exons 18, 19, 20, and 21 of the EGFR gene were found.

LPF-NT is the most difficult to identify. Agaram et al. first reported LPF-NT in 2016 as a rare, soft tissue tumor that occurs mainly in the trunk and extremities of children and young adults. The tumor consists of CD34-positive spindle cells, similar to the infiltrative growth pattern of lipofibromatosis. The two are distinguished by positive S-100 protein expression and NTRK1 gene rearrangement in LPF-NT [18]. Wang et al. suggested that CD34 and S-100 positivity, diffuse positivity for NTRK1 and panTRK in immunohistochemistry, and NTRK1 gene rearrangement detection could help in the diagnosis [8]. In contrast, lipofibromatosis was mostly negative for S-100 and panTRK [18]. Therefore, immunohistochemical and FISH results for these indices were purposely shown to demonstrate that the lesion was not an LPF-NT.

In conclusion, a case of lipofibromatosis with an atypical clinical presentation and confusing history, appearance, and location was reported. Thus, for such a large or rapidly enlarging lesion, depth and extent can be confirmed by imaging first, and the diagnosis can be made by biopsy. This also suggests that the diagnostic process of lipofibromatosis can be further improved, and easier and faster methods can be developed to help physicians detect and intervene early.

## Data Availability

All data generated or analysed during this study are included in this published article [and its supplementary information files].

## Abbreviations

FISH: Fluorescence in situ hybridization
SMA: Smooth muscle actin
LPF-NT: Lipofibromatosis-like neural tumor
FHI: Fibrous hamartoma of infancy
IH: Infantile hemangioma
TA: Tufted angioma
KHE: Kaposiform hemangioendothelioma
HE: Hematoxylin and eosin
